# Analyzing How Skinfold Thickness Affects Log-Transformed EMG Amplitude–Power Output Metrics

**DOI:** 10.3390/bioengineering11121294

**Published:** 2024-12-20

**Authors:** Matthew J. Kraydich, Jacob Gonzalez, Marcus A. Ziebold, Patrick N. Asmar, Amanda Chehab, Moh H. Malek

**Affiliations:** 1 Physical Therapy Program, Department of Health Care Sciences, Wayne State University, College of Pharmacy and Health Sciences, Detroit, MI 48201, USA; 2 Integrative Physiology of Exercise Laboratory, Department of Health Care Sciences, Wayne State University, College of Pharmacy and Health Sciences, Detroit, MI 48201, USA

**Keywords:** electromyography, signal processing, individual differences, adipose tissue

## Abstract

Background: The purpose of this study was to determine whether accounting for skinfold thickness would reduce the variability observed on a subject-by-subject basis for the *y*-intercept and slope terms derived from the log-transformed EMG amplitude–power output relationship. We hypothesized that using skinfold thickness as a covariate would reduce the subject-by-subject variability in the *y*-intercept and slope terms and, therefore, indicate potential mean differences between muscle groups. Methods: Subjects had the skinfold from their three superficial quadriceps femoris muscles measured and then EMG electrodes placed over the three muscles. Thereafter, each subject performed an incremental single-leg knee-extensor ergometer exercise test to voluntary exhaustion. Results: The results indicated that using skinfold thickness as a covariate did not change the statistical outcome when comparing the mean values for the *y*-intercept or slope terms across the three superficial quadriceps femoris muscles. Conclusion: These findings suggest that there may be other factors that are influencing the subject-by-subject variability for the *y*-intercept and slope terms, respectively.

## 1. Introduction

One of the hallmarks of assessing neuromuscular fatigue is the use of electromyography (EMG). The use of surface EMG is typically used for large muscle groups such as the quadriceps femoris muscles [1], whereas fine wire or needle EMG is used for small muscle groups such as those in the finger [1]. Nevertheless, in the majority of studies, the EMG amplitude is used as an indicator of muscle activation (i.e., recruitment and/or firing rating) for a given movement [2,3]. For continuous muscle action such as those associated with running or cycling, studies have shown that the EMG amplitude has high intersession reliability [2].

The analyses of the patterns of responses between the EMG amplitude versus torque (or power) output have been assessed using polynomial regression [4]. To this extent, the relationship may be described as linear, quadratic, or cubic [4]. However, a limitation of this approach is that comparisons across groups cannot be assessed. That is, comparing the EMG amplitude between different muscle groups (i.e., vastus lateralis versus rectus femoris) or the left leg versus the right leg [4]. To overcome this statistical limitation, Herda and colleagues [5,6,7] developed the log-transformed EMG amplitude–power output relationship. The utility of this model is that for each subject a *y*-intercept and slope term is generated from the EMG amplitude versus torque (or power) relationship [5,6,7]. As a result, inferential statistics can be used to make comparisons between groups or muscles [5,6,7].

The log-transformed EMG amplitude–power output relationship has high reliability for continuous muscle action for the *y*-intercept and slope term [8]. However, when comparing the *y*-intercept on a subject-by-subject basis there is large variability between subjects [4,8,9,10,11]. A similar observation has been seen with the slope term [4,8,9,10,11]. This issue may, in part, be accounted for the lack of statistical significance reported when comparing *y*-intercept or slope terms across different groups within a single study [4,8,9,10,11].

One potential explanation for the high inter-subject variability may be associated with the skinfold thickness of the subjects. That is, since the EMG signal is recorded from electrodes placed on the surface of the thigh, the subcutaneous fat acts as a low-bandpass filter [12,13]. Studies have characterized subcutaneous fat as being a poor conductor of electric current and possessing high electrical resistance even though they provide a type of insulation for tissues and organs [14,15,16]. For example, Petrofsky [17] electrically stimulated the rectus femoris muscle of subjects and found a strong positive correlation (r = 0.95; *p <* 0.001) between the amount of subcutaneous fat layer and loss of the EMG signal. The author concluded that individuals with higher skinfold thickness (i.e., thicker layer of fat) had a slower resistor capacitor time constant (i.e., the time it takes for the capacitor to charge to 63.2% of its full value), and therefore, less transfer of energy to the electrode [17]. De la Barrera and Milner [12] used an array of surface EMG over the muscle of the upper arm in both men and women to determine the influence of skinfold thickness on the EMG signal. The investigators reported that EMG signal deteriorated with a concomitant increase in subcutaneous fat [12]. Similarly, Baniqued et al. [18] reported that higher subcutaneous fat significantly influenced the mean power frequency at the fatigue threshold. Moreover, Zaheer and colleagues [19] reported that for some muscles there is a negative correlation between skinfold thickness and the signal-to-noise ratio for surface EMG. Therefore, since skinfold thickness may vary on a subject-by-subject basis, this variable needs to be considered as a potential covariate for any inferential statistics comparing the *y*-intercept or slope terms across different groups. 

The purpose of this study, therefore, was to determine whether accounting for skinfold thickness would reduce the variability observed on a subject-by-subject basis for the *y*-intercept and slope terms. We hypothesized that using skinfold thickness as a covariate would reduce the subject-by-subject variability in the *y*-intercept and slope terms and, therefore, indicate potential mean differences between muscle groups.

## 2. Materials and Methods

### 2.1. Experimental Design and Approach

Each subject visited the laboratory on a single occasion. During this visit, the subject underwent skinfold measurements as well as the incremental single-leg knee-extensor ergometer test. Moreover, surface EMG electrodes were placed on the three superficial quadriceps femoris muscles of the non-dominant limb which was the leg used to perform the exercise test.

### 2.2. Subjects

An a priori power analysis with α = 0.05 and *β* = 0.10 with an effect size of 1.10 based on previous studies indicated a minimum of 9 subjects. Therefore, 13 healthy college-aged men ranging from 23 to 32 years old (Table 1) volunteered as subjects for the present study. All subjects were instructed not to exercise the day (24 h) prior to their visit. In addition, due to the potential confounding variable of hydration status on the EMG signal, subjects were instructed to drink adequate fluids 24 h prior to their visit as recommended by the American College of Sports Medicine [20]. During the testing day, subjects were instructed to refrain from caffeine consumption [11,21,22,23,24,25]. All procedures were approved by the University Institutional Review Board for Human Subjects, and each subject signed an informed consent form.

### 2.3. Incremental Single-Leg Knee-Extensor Ergometer

As previously described [11,21,22,23,24,25], the single-leg knee-extensor ergometer exercise paradigm allows for isolation of the quadriceps femoris muscles during continuous muscle action. As a result, respiratory and cardiovascular limitations to incremental exercise such as those inherited from treadmill running or cycle ergometry are attenuated [26]. Briefly, the subject was seated in a semirecumbent position with the non-dominant leg (based on kicking preference) in a modified McKesson Standard Walker Boot that was connected to the crank handle of a cycle ergometer [11,21,22,23,24,25]. The dominant leg, therefore, was resting on a platform. Illustrations of this device have been previously published [2,22]. Each subject was instructed to perform a kicking motion in which the leg was extended from 90° to an ending position of 170° [11,21,22,23,24,25]. 

Once the subject was ready to begin the exercise test, they performed a 2 min warm-up at 4 W maintaining a kick cadence of ~70 revolutions per minute. The power output was then increased to 4 W/min until the subject was unable to maintain the kick cadence despite strong verbal encouragement. In addition, the subject wore a Polar heart rate monitor to determine peak heart rate at the end of the exercise test [11,21,22,23,24,25]. Moreover, the subject was asked their Rating of Perceived Exertion (RPE) using the Modified Borg Scale (0–10) throughout to the exercise workout [11,21,22,23,24,25]. 

### 2.4. Placement of EMG Electrodes

Three separate bipolar (20 mm center-to-center) surface electrodes (EL500-6, BIOPAC Systems, Inc., Santa Barbara, CA, USA) were placed over the vastus lateralis, rectus femoris, and vastus medialis muscles. The specific measurement and identification of the location to place each electrode was consistent with the recommendations of SENIAM (Surface ElectroMyoGraphy for the Non-Invasive Assessment of Muscles) [27]. In addition, a reference electrode was placed over the iliac crest [11,21,22,23,24,25]. At each electrode site, the skin was carefully shaved and abraded with sandpaper, then cleaned with 70% isopropyl alcohol. The interelectrode impedance was kept below 2000 ohms, consistent with our previous work [11,21,22,23,24,25]. The EMG signal from each electrode placement site was amplified (gain: ×1000) using differential amplifiers (EMG 100B, BIOPAC Systems, Inc., Santa Barbara, CA, USA).

### 2.5. EMG Signal Acquisition and Processing

The raw EMG signals were digitized at 1000 Hz and stored in a personal computer (Dell Inspiron E1705, Dell Inc., Round Rock, TX, USA) for subsequent analysis. All signal processing was performed using custom programs written with LabVIEW programming software (version 2019, National Instruments, Austin, TX, USA). The EMG data were collected at the last 10 s of each stage. The EMG signals were bandpass filtered (fourth-order Butterworth) at 10–500 Hz. The amplitude value for each stage was calculated for each subject based on the average of all the completed bursts during the sampling window [11,21,22,23,24,25].

### 2.6. Skinfold Measurements

After the measurement of the EMG electrode sites, skinfold thickness was assessed using Lange^®^ calipers (Model 68902; Beta Technology Inc., Cambridge, MD, USA). Three measurements were taken at each electrode placement site for the three superficial quadriceps femoris muscles. The measurements were performed by a single individual with experience in skinfold assessments. For each site, if the first two measurements were within 0.5 mm, then the third measurement was not taken. Alternatively, if the first two measurements were outside the 0.5 mm range, then the third measurement was taken. The average of the measurements for each site were taken for each subject.

### 2.7. Statistical Analysis

The absolute EMG amplitude was used for the following analyses rather than normalizing the EMG amplitude (i.e., percentage of maximal power output). The data were analyzed using the log-transformed model of Herda et al. [7] and previously used by our laboratory [4,8,11]. Briefly, the absolute EMG amplitude (µVrms) values were used. Briefly, the linear regression model was transformed to a natural log as represented by the equation:

ln[*Y*] = *b*(ln[*X*]) + ln[*a*] (1)

where ln[*Y*] is the natural log of the EMG amplitude values, ln[*X*] is the natural log of the power output, *b* is the slope term, and ln[*a*] is the natural log of the ‘*a*’ term [4,7,8,11]. In addition, by using an antilogarithm function, Equation (1) is converted to

*Y* = *aX*^*b*^ (2)

where the exponent *Y* is equal to the predicted EMG amplitude, *X* is the power output, and *b* is the slope of the log-transformed equation [4,7,8,11]. Therefore, using Equation (2), values such as the coefficient of determination (R^2^), slope, and *y*-intercept were calculated on a subject-by-subject basis. 

All data presented in the present study are mean ± SEM (standard error of the mean) values. For the determination of mean differences between the dependent variables, separate one-way repeated measures ANCOVAs (Analysis of Covariance) with skinfold thickness as the covariate for the *y*-intercept and slope terms were conducted. All statistical analyses were performed using the Statistical Package for the Social Sciences software (v. 28.0, IBM SPSS, Armonk, NY, USA) with an alpha level set at *p* ≤ 0.05.

## 3. Results

### 3.1. Comparison of a Terms Across Muscles with and Without Covariate

**Figure 1:**
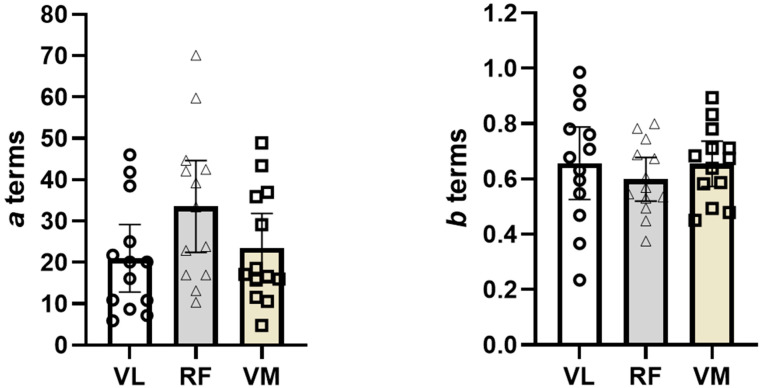
Plotted individual values as well as means ± 95% confidence intervals for the *a* and *b* terms, respectively.

Table 1 and Table 2 represent the anthropometric data and the subject-by-subject values for the *a* and *b* terms, respectively. When performing the one-way repeated measures ANOVA, we did not meet the sphericity assumption (*p <* 0.001); therefore, we used the Greenhouse–Geisser correction for the formal analyses. Thus, the one-way repeated measures ANOVA for the *a* term revealed no significant mean differences [F(1.1,13.7) = 3.31; *p >* 0.05] between the three superficial quadriceps muscles (mean ± SEM: VL: 21 ± 4; RF: 33 ± 5; VM: 23 ± 4). We also plotted individual values as well as means ± 95% confidence intervals for the *a* term (Figure 1). 

We then performed the same analyses; however, we used the rectus femoris skinfold as the covariate. Similarly, we did not meet the sphericity assumption (*p =* 0.002); therefore, we used the Greenhouse–Geisser correction for the formal analyses. Thus, the one-way repeated measures ANCOVA for the *a* term revealed no significant mean differences [F(1.1,12.8) = 0.620; *p* > 0.05] between the three superficial quadriceps muscles (adjusted mean ± SEM: VL: 21 ± 4; RF: 33 ± 5; VM: 23 ± 4).

### 3.2. Comparison of b Terms Across Muscles with and Without Covariate

When performing the one-way repeated measures ANOVA, we did meet the sphericity assumption (*p* = 0.21). Thus, the one-way repeated measures ANOVA for the *b* term revealed no significant mean differences [F(2,24) = 0.870; *p* > 0.05] between the three superficial quadriceps muscles (mean ± SEM: VL: 0.65 ± 0.06; RF: 0.59 ± 0.04; VM: 0.66 ± 0.4). We also plotted individual values as well as means ± 95% confidence intervals for the *b* term (Figure 1).

We then performed the same analyses; however, we used the rectus femoris skinfold as the covariate. When performing the one-way repeated measures ANCOVA, we did meet the sphericity assumption (*p* = 0.12). Thus, the one-way repeated measures ANCOVA for the *b* term revealed no significant mean differences [F(2,22) = 1.93; *p* > 0.05] between the three superficial quadriceps muscles (adjusted mean ± SEM: VL: 0.66 ± 0.06; RF: 0.60 ± 0.03; VM: 0.66 ± 0.4).

## 4. Discussion

The principle finding of the current investigation was that skinfold thickness, as a covariate, did not result in significant mean differences for the *y*-intercept (*a* term) or slope (*b* term) between the three superficial quadriceps femoris muscles for the log-transformed EMG amplitude–power output relationship. Nevertheless, when examining the *b* term for each muscle on a subject-by-subject basis, the majority (38 out of 39) were significant based on the 95% confidence interval (Table 2). To our knowledge, this is the first study which has examined skinfold thickness as a covariate to minimize the subject-by-subject variability for the *a* term and *b* term.

### 4.1. y-Intercept (a Term)

One of the components of the log-transformed EMG amplitude–power output relationship equation is the *y*-intercept or *a* term. Traditionally in linear regression, the *y*-intercept is the point at which the line of best fit (i.e., regression line) crosses the *y*-axis [10]. The *y*-intercept, however, in the log-transformed EMG amplitude–power output relationship is always positive, with the change in the *y*-intercept representing an upward or downward shift in the EMG amplitude versus power output relationship [4,8,11]. Therefore, the *a* term is determined on a subject-by-subject basis which, in turn, allows for examining potential changes in mean differences between muscles (Table 2) or other independent variables [4,8,11]. Herda et al. [5] has reported that the *a* term in the log-transformed EMG amplitude–power output relationship may be impacted by skinfold thickness. As such, the investigators compared and contrasted between aerobically and resistance-trained relative to a sedentary cohort [5]. As expected, the mean skinfold thickness for the aerobically trained cohort was significantly lower than the sedentary cohort. Moreover, the investigators found that the mean *a* term for the aerobically trained cohort was significantly different relative to the resistance-trained and sedentary cohort [5].

It has been suggested that the *a* term is associated with changes in the upward or downward shifts in the EMG amplitude versus power output across the entire across test [4,7,8,11]. Therefore, differences inherit to each subject (i.e., skinfold thickness) may influence the *a* term [4,7,8,11]. The rationale for the potential influence of skinfold thickness on the *a* term resides in the approach used to record the EMG signal. Briefly, electrodes are placed on the surface of the skin which overlays the target muscle. The subcutaneous fat, therefore, may behave as a low-pass filter of the EMG signal prior to being recorded [5]. Therefore, the differences in skinfold thickness on a subject-by-subject basis may be a potential confounding variable when using the *a* term as a dependent variable. Indeed, studies have shown that skinfold thickness can influence the recording of the EMG activity [12,28]. For example, Nordander and colleagues [28] examined the impact of skinfold thickness of the trapezius muscle on the EMG amplitude in 12 middle-aged women during isometric muscle action. The investigators reported that skinfold thickness accounted for a significant part of the subject-by-subject variability in the EMG amplitude [28]. The present study attempted to address this issue by measuring skinfold thickness in group of healthy college-aged men and use this information as a covariate to determine potential changes in mean values for the *a* term across the three superficial quadriceps femoris muscles. As such, we used the skinfold thickness for the rectus femoris muscle as the covariate to control for differences in skinfold thickness on subject-by-subject basis. The rationale was based on the previous studies [29], indicating that the single-leg knee-extensor ergometer engages the rectus femoris muscle primarily as opposed to the other superficial quadriceps femoris muscles. Nevertheless, we found that when the skinfold thickness for the rectus femoris muscle was used as a covariate, it did not result in any significant mean differences between the *a* terms for the superficial quadriceps femoris muscles. That is, accounting for the subject’s skinfold thickness did not influence the mean value for the *a* terms across the three muscles. One potential explanation may be that we did not have a homogenous sample relative to skinfold thickness. That is, unlike the study by Herda et al. [5], we did not have specific groups (i.e., aerobically trained) based on habitual exercise history which would result in a more homogeneous skinfold thickness.

### 4.2. Slope (b Term)

The *b* term in a regression model generally represents the rate of change in the dependent variable for each unit of change in the predictor variable [4,7,8,11]. It has been suggested that the *b* term is associated with changes in motor unit recruitment of the exercising muscle(s) from a biological perspective [4,7,8,11]. Thus, in the context of the EMG amplitude–power output relationship, a *b* term value greater than 1 suggests an acceleration, while a *b* term value less than 1 indicates deceleration [4,7,8,11]. A number of studies have examined the *b* term in the log-transformed EMG amplitude–power output relationship. For example, Noble and colleagues [11] examined potential differences between the *b* term for the superficial quadriceps femoris muscles performing a single-leg knee-extensor ergometer versus single-leg cycle extensor ergometer using a within-subjects research design. The authors reported no significant exercise mode by muscle interaction for the *b* term [11]. Eason et al. [8] reported a high intraclass correlation for the *a* term (r = 0.79) and *b* term (r = 0.78) for the rectus femoris muscle for incremental single-leg knee-extensor ergometer. These data, therefore, suggest that the two indices are highly reliable [8]. In addition, when examining the *b* term in the Eason et al. [8] study, variability in the values exist on a subject-by-subject basis. Boccomino et al. [9] reported no significant mean differences for the *b* term between the nondominant and dominant leg for the double-leg knee-extensor ergometer; however, similarly to other studies, there was variability in the *b* term value from subject-to-subject. In the present study, we also observed a wide range of *b* terms for the vastus lateralis (0.404 to 0.985), rectus femoris (0.651 to 0.977), and vastus medialis (0.643 to 0.933), respectively. Moreover, our hypothesis that skinfold thickness may account for the variability observed on a subject-by-subject basis was not supported by the statistical analyses. 

## 5. Conclusions

In conclusion, the findings for the current study suggest that skinfold thickness did not reduce the subject-by-subject variability in the *y*-intercept and slope term indices derived from the log-transformed EMG amplitude–power output relationship. Our study, however, did find that the slope term for the majority of subjects across the three superficial quadriceps femoris muscles were within the 95% confidence interval. Thus, future studies may need to examine other factors that may influence the EMG signal, such as the hydration status of the subject.

## Figures and Tables

**Table 1:** Subject anthropometrics measurements.

Index	Mean ± SEM
Age (y)	25.5 ± 0.7
Height (m)	1.84 ± 0.03
Body mass (kg)	87.0 ± 3.2
Vastus lateralis skinfold thickness (mm)	35.7 ± 2.1
Rectus femoris skinfold thickness (mm)	36.1 ± 2.2
Vastus medialis skinfold thickness (mm)	33.2 ± 2.2

**Table 2:** Results of the *y*-intercept (*a* term) and slope (*b* term) on a subject-by-subject basis.

EMG Amplitude (Log Transformed)									
	Vastus Lateralis			Rectus Femoris			Vastus Medialis		
Subject	*R^2^*	*a*	*b* (95% CI)	*R^2^*	*a*	*b* (95% CI)	*R^2^*	*a*	*b* (95% CI)
1	0.404	41.824	0.234 (−0.0498 to 0.518)	0.825	22.920	0.494 (0.267 to 0.721)	0.784	35.874	0.479 (0.228 to 0.731)
2	0.814	46.063	0.469 (0.269 to 0.670)	0.772	44.701	0.448 (0.230 to 0.666)	0.826	43.380	0.450 (0.265 to 0.635)
3	0.833	5.930	0.918 (0.645 to 1.19)	0.809	33.448	0.605 (0.410 to 0.800)	0.859	11.588	0.780 (0.571 to 0.990)
4	0.824	10.913	0.631 (0.438 to 0.825)	0.906	16.945	0.545 (0.429 to 0.661)	0.895	4.759	0.832 (0.643 to 1.02)
5	0.950	21.758	0.677 (0.522 to 0.832)	0.977	42.521	0.673 (0.571 to 0.775)	0.893	15.800	0.893 (0.491 to 1.01)
6	0.738	8.671	0.868 (0.583 to 1.15)	0.744	10.381	0.688 (0.465 to 0.910)	0.712	16.610	0.638 (0.414 to 0.861)
7	0.894	16.119	0.780 (0.512 to 1.05)	0.950	23.807	0.799 (0.616 to 0.981)	0.880	18.541	0.673 (0.425 to 0.922)
8	0.777	20.086	0.595 (0.355 to 0.837)	0.672	59.740	0.534 (0.253 to 0.815)	0.643	15.959	0.582 (0.254 to 0.909)
9	0.693	25.028	0.547 (0.354 to 0.740)	0.651	39.252	0.569 (0.349 to 0.790)	0.735	29.079	0.586 (0.399 to 0.772)
10	0.712	20.086	0.707 (0.340 to 1.07)	0.827	13.197	0.744 (0.467 to 1.02)	0.710	36.966	0.710 (0.352 to 1.07)
11	0.985	7.171	0.985 (0.769 to 0.927)	0.685	70.105	0.375 (0.183 to 0.566)	0.933	10.591	0.683 (0.545 to 0.822)
12	0.842	10.805	0.760 (0.528 to 0.991)	0.712	16.945	0.782 (0.431 to 1.13)	0.826	17.116	0.711 (0.481 to 0.941)
13	0.670	38.475	0.366 (0.070 to 0.662)	0.895	42.098	0.530 (0.321 to 0.739)	0.846	48.911	0.493 (0.251 to 0.735)

## Data Availability

All data from this study are available from the authors upon reasonable request.
